# The Primary Result of Prospective Randomized Multicenter Trial of New Spray-Type Bio-absorbable Adhesion Barrier System (TCD-11091) Against Postoperative Adhesion Formation

**DOI:** 10.1007/s11605-017-3503-1

**Published:** 2017-07-25

**Authors:** Takeshi Suto, Masahiko Watanabe, Takeshi Endo, Koji Komori, Masayuki Ohue, Yukihide Kanemitsu, Masaaki Itou, Yasumasa Takii, Toshimasa Yatsuoka, Manabu Shiozawa, Tetsushi Kinugasa, Hideki Ueno, Tadatoshi Takayama, Tadahiko Masaki, Hiroyuki Masuko, Hisanaga Horie, Masafumi Inomata

**Affiliations:** 1 Yamagata Prefectural Central Hospital, 1800 Aoyagi, Yamagata, Yamagata Japan; 2 Department of Surgery, Kitasato University School of Medicine, Kanagawa, Japan; 3 Department of Surgery, Hasuda Hospital, Saitama, Japan; 4 Department of Gastroenterological Surgery, Aichi Cancer Center Hospital, Aichi, Japan; 5 Department of Surgery, Osaka Medical Center for Cancer and Cardiovascular Diseases, Osaka, Japan; 6 Department of Colorectal Surgery, National Cancer Center Hospital, Tokyo, Japan; 7 Department of Colorectal Surgery, National Cancer Center Hospital East, Chiba, Japan; 8 Department of Surgery, Niigata Cancer Center Hospital, Niigata, Japan; 9 General and Gastroenterological Surgery, Showa University Fujigaoka Hospital, Kanagawa, Japan; 10 Department of Gastrointestinal Surgery, Kanagawa Cancer Center, Kanagawa, Japan; 11 Department of Surgery, Kurume University School of Medicine, Fukuoka, Japan; 12 Department of Surgery, National Defense Medical College, Saitama, Japan; 13 Department of Digestive Surgery, Nihon University School of Medicine, Tokyo, Japan; 14 Department of Surgery, School of Medicine, Kyorin University, Tokyo, Japan; 15 Department of Surgery, Nikko Memorial Hospital, Hokkaido, Japan; 16 Department of Surgery, Jichi Medical University, Tochigi, Japan; 17 Department of Gastroenterological and Pediatric Surgery, Oita University Faculty of Medicine, Oita, Japan

**Keywords:** Randomized-control trial, Adhesions, Rectal cancer, Sprayer, Adspray

## Abstract

**Background:**

Postoperative adhesions are the major cause of postoperative complications including intestinal obstruction, infertility, and chronic pelvic pain. In order to reduce postoperative adhesions, Terumo Corporation (Tokyo, Japan) has developed an adhesion barrier system (TCD-11091) which is easy to use at the treatment site in various surgical procedures including laparoscopic surgeries. We conducted a prospective randomized single-blind study in patients who underwent laparotomy with ileostomy.

**Methods and Results:**

One hundred twenty-six patients were randomly assigned to TCD-11091 group (*n* = 62) or non-treatment group (*n* = 62). Patient backgrounds were similar between the groups. At the time of ileostomy closure (the second-look surgery), the observation was performed on 55 in the TCD-11091 group and 43 in the control group. The incidence of adhesions observed at the second-look surgery was significantly lower in the TCD-11091 group (52.7 versus 90.7%; *p* < 0.001). For the secondary endpoints, the incidence of wide extent adhesions (grade 2 or higher) was significantly reduced (38.2 versus 79.1%; *p* < 0.001). Regarding the severity of adhesions, the incidence of grade 2 or higher adhesions was also significantly lower in the TCD-11091 group (47.3 versus 88.4%; *p* < 0.001). No differences in the incidence of adverse events were found between the TCD-11091 group and the non-treatment group (85.2 versus 75.4%; *p* = 0.225).

**Conclusions:**

Use of TCD-11091 was safe and associated with significantly lower incidence of adhesion and severity of adhesions compared with non-treatment procedure.

## Introduction

Postoperative adhesions are a common consequence of surgery where the organs originally isolated from each other bind to each other during the repair process of the living tissues that were impaired or injured at the time of the surgical procedure. Postoperative adhesions can occur regardless of the type of procedure in any surgical area including the abdominal cavity or pelvic cavity; the frequency is known to be 55 to 95%.^1^ Adhesions are the major cause of postoperative complications including intestinal obstruction, infertility, and chronic pelvic pain.

In order to reduce postoperative adhesions, Terumo Corporation (Tokyo, Japan) has developed an adhesion barrier system which is easy to use at the treatment site in various surgical procedures including laparoscopic surgeries. The ADBEE study of TCD-11091 was conducted in the EU.^2^ In the ADBEE study, 32 patients scheduled to undergo laparoscopic myomectomy were randomized in a 2:1 ratio to either the TCD-11091 group or the non-treatment group, and the adverse events over a 28-day postoperative period were assessed. The adverse events during this period were 62% in the TCD-11091 group and 81% in the non-treatment group (*p* = 1.000). In the TCD-11091 group, no adverse events related to TCD-11091 were noted. Regarding the occurrence of adverse events, neither a significant difference between the groups nor adverse events related to TCD-11091 was noted. Therefore, the safety of TCD-11091 was demonstrated by the ADBEE study. Based on these results, we conducted a prospective randomized single-blind study where a non-treatment group served the control, to confirm the efficacy and safety of TCD-11091 as an inhibitory effect of postoperative adhesion formation in patients who underwent laparotomy with ileostomy.

## Method

### Study Design

This study was designed as a prospective, multicenter, randomized (in a 1:1 ratio) single-blind study using a non-treatment group as the control, and enrolled 124 patients.

### Study Device

TCD-11091 consists of a polymer kit and sprayer kit. To apply TCD-11091, powder A solution (NHS-modified carboxymethyl dextrin) and B solution (sodium carbonate/sodium hydrogen carbonate) are prepared with the polymer kit and loaded into a double syringe, which is attached to the nozzle. The two solutions are mixed uniformly with the assistance of compressed medical air at the nozzle tip and sprayed in the form of mist. The two sprayed solutions gelate rapidly over the living tissues. The gel adheres to the injury site and forms a physical barrier to provide the adhesion prevention effect. After serving its purpose, the gel gradually degrades, is absorbed, and is then excreted, mainly into urine. The sprayer of this system was designed to enable the abdominal cavity to be accessed through a 5-mm trocar in line with its use during laparoscopy. Consequently, the formed gel contains microbubbles and appears white. Therefore, the surgeon can visually confirm the extent of gel coverage through the laparoscope.

### Study Method

This study was approved by the institutional review board (IRB) at each participated institutes. All patients signed the informed consent form. Subjects were patients with primary rectal cancer who were scheduled to undergo temporary ileostomy. Rectal resection and ileostomy (the initial surgery) through midline laparotomy and ileostomy closure (the second-look surgery) were performed. Patients were allocated at the time of enrollment in the study after informed consent was obtained. Randomized allocation was performed according to the minimization method by the dynamic allocation and using clinical site as allocation factor. Then, the initial surgery was performed according to the allocation. At the second-look surgery, laparoscopic video recordings of the area under the midline incision were made in both groups. For the efficacy evaluation, based on the laparoscopic images of the abdominal cavity at the time of the second-look surgery, the presence, extent, and severity of adhesions were assessed by the efficacy assessment committee which was independent of the study site and the sponsor. For the initial surgery, the operation was performed under general anesthesia according to the standard practice of each site. At the end of the surgery, TCD-11091 was sprayed to fully cover the organs under the midline incision, and pictures were taken from directly above this incision. After pictures were taken, the peritoneum was sutured with an absorbable synthetic monofilament suture to close the abdomen. Taking pictures of adhesions through the laparoscope at the second-look surgery was an essential part of the efficacy evaluation in this study, and variations in resolution were minimized by employing a photographic method that was standardized across all sites. Movies were produced laparoscopically so that the upper edge (cranial end) and the lower edge (caudal end) of the midline incision were included in the images and the location of the midline incision could be confirmed. The independent efficacy assessment committee was established for determination of adhesions and assessed the presence, extent, and severity of adhesions. The extent and severity of adhesions under the midline incision were assessed on a 4-grade scale (Tables 1 and 2).

**Table 1:** Grade classification of extent of adhesions

Grade 0	None
Grade 1	Adhesions <1/3 the length of the midline incision
Grade 2	Adhesions between 1/3 and <2/3 the length of the midline incision
Grade 3	Adhesions ≥2/3 the length of the midline incision

**Table 2:** Grade classification of severity of adhesions

Grade 0	None
Grade 1	Film-like with no neovascularization
Grade 2	Moderately thick with partial neovascularization
Grade 3	Thick, solid adhesions with neovascularization

### Inclusion/Exclusion Criteria

Major inclusion criteria were the following: 20 years and older, scheduled to undergo the initial surgery and to undergo the second-look surgery 3 to 6 months after the initial surgery, able to undergo the first- and second-look surgeries under general anesthesia, and indicated for laparotomy. Major exclusion criteria were active cancer other than rectal cancer, history of radiotherapy in the abdominal cavity or pelvic cavity, peritonitis, severe diabetes or abnormal glucose metabolism, glycogenosis, serious liver disorder, and serious renal disorder. All inclusion and exclusion criteria are summarized in Table 3.

**Table 3:** Inclusion and exclusion criteria for the trial

Inclusion criteria	Exclusion criteria
1. Patients scheduled to undergo the initial surgery, and to undergo the second-look surgery 3 to 6 months after the initial surgery 2. Patients able to undergo the first- and second-look surgeries under general anesthesia 3. Patients indicated for laparotomy 4. Patients with ECOG performance status of 0 or 1 5. Patients aged 20 years or older at the time the consent form is signed 6. Patients able to undergo all scheduled observations and who consented to all details of the observations 7. Patients able to comply sufficiently with the procedures and regulations in the study 8. Patients able to freely provide written informed consent to participate in the study using the consent form approved by the IRB of each site	1. A history of hypersensitivity to substances derived from corn starch 2. Patients with active cancer other than rectal cancer 3. A history of radiotherapy in the abdominal cavity or pelvic cavity 4. A history of surgery in the abdominal cavity or pelvic cavity accompanied by a laparotomy scar (excluding surgery for appendicitis) 5. Patients with peritonitis 6. Patients subject to emergency surgery 7. Patients with severe diabetes or abnormal glucose metabolism 8. Patients with glycogenosis 9. Patients with serious liver disorder 10. Patients with serious renal disorder 11. Patients with a BMI of >30 12. Patients for whom chronic corticosteroid treatment is necessary 13. Patients scheduled for another laparotomy in the period between the initial surgery and second-look surgery 14. Laparoscopic assessment of adhesions may not be performed safely at the second-look surgery 15. Patients who participated in other clinical studies within 6 months prior to the day of signing the consent form, or patients who are currently participating in other clinical studies 16. Pregnant or lactating patients 17. Patients that the investigator or sub-investigator consider inappropriate for inclusion in the study as they have tested positive for infections, such as hepatitis B virus, hepatitis C virus, and human immunodeficiency virus 18. Patients with medical conditions that may hinder the safety or efficacy evaluation of the study device 19. Patients the investigator or sub-investigator considered inappropriate for inclusion in the study for any other reason

### Endpoints

The primary endpoint of this study was the incidence of adhesions under the midline incision and was defined as “the number of patients who are determined to have adhesions/the number of patients who underwent the second-look surgery.” The secondary endpoints included the following: (1) the extent of adhesions under the midline incision, (2) the severity of adhesions under the midline incision, (3) major adverse events (MAEs: intestinal obstruction, abdominal abscess, peritonitis, postprocedural site wound infection), (4) serious adverse events, (5) adverse events, (6) adverse events which occur from the initiation of the initial surgery to the day of discharge after the second-look surgery and for which a causal relationship to TCD-11091 cannot be ruled out, and (7) malfunction of the study device.

### Study Organization

This study was conducted by Terumo Corporation (Tokyo, Japan), and the study management and monitoring and source document verification of all data, statistical analyses, and data management were conducted by A2 Healthcare Corporation (Tokyo, Japan), the CRO. An independent efficacy assessment committee was established for determination of adhesion status and assessed the presence, extent, and severity of adhesions. Safety data and important efficacy endpoints were evaluated at appropriate intervals. The data safety monitoring board (DSMB) was established for the purpose of recommending continuation or discontinuation of the study or changes in the study to the sponsor.

### Statistical Analysis

To calculate the sample size to be enrolled in this study, the incidence of adhesions was estimated to be 80% or higher in the control group based on the results of the preceding five studies (86.2 to 100%).^3^–^8^ The incidence of adhesions was estimated to be reduced to 50% when TCD-11091 was used based on the report of the study conducted by Becker et al., which had the largest sample size among the clinical studies of Seprafilm.^8^ Therefore, the incidence of adhesions with the study device was set at 50%. Based on the above, the incidences of adhesions with and without the study device were assumed to be 50 and 80%, respectively. The number of patients required for a group was calculated to be 52 patients with a power of 90%. Consequently, the planned sample size per group was set at 62, and the total sample size required for this study was set at 124 based on the assumption that 15% of the patients might drop out. In the statistical analyses, summary statistics including mean, standard deviation, minimum, median, and maximum were calculated. Regarding patient background, comparisons were conducted between groups: for continuous values, the *t* test was used to compare differences in the mean values; for ordered categorical variables, the Wilcoxon rank sum test was used to compare differences in the median values; and for nominal variables, the chi-squared test was used to assess differences in the frequencies. The incidences of adhesions, adverse events, and MAEs were tested using Fisher’s exact test, and the extent and severity of adhesions were tested by between-group comparisons using the Wilcoxon rank sum test. The two-sided significance level was set at 5%. Study data were collected as EDC using DATATRAK ONE (version 13.2.5.; DATATRAK International, OH, USA) and analyzed using SAS (version 9.2; SAS Institute, Cary, NC, USA). The incidence of adhesions, the primary endpoint, was compared using Fisher’s exact test, and the extent and severity of adhesions were compared using the Wilcoxon rank sum test.

## Results

**Fig. 1:**
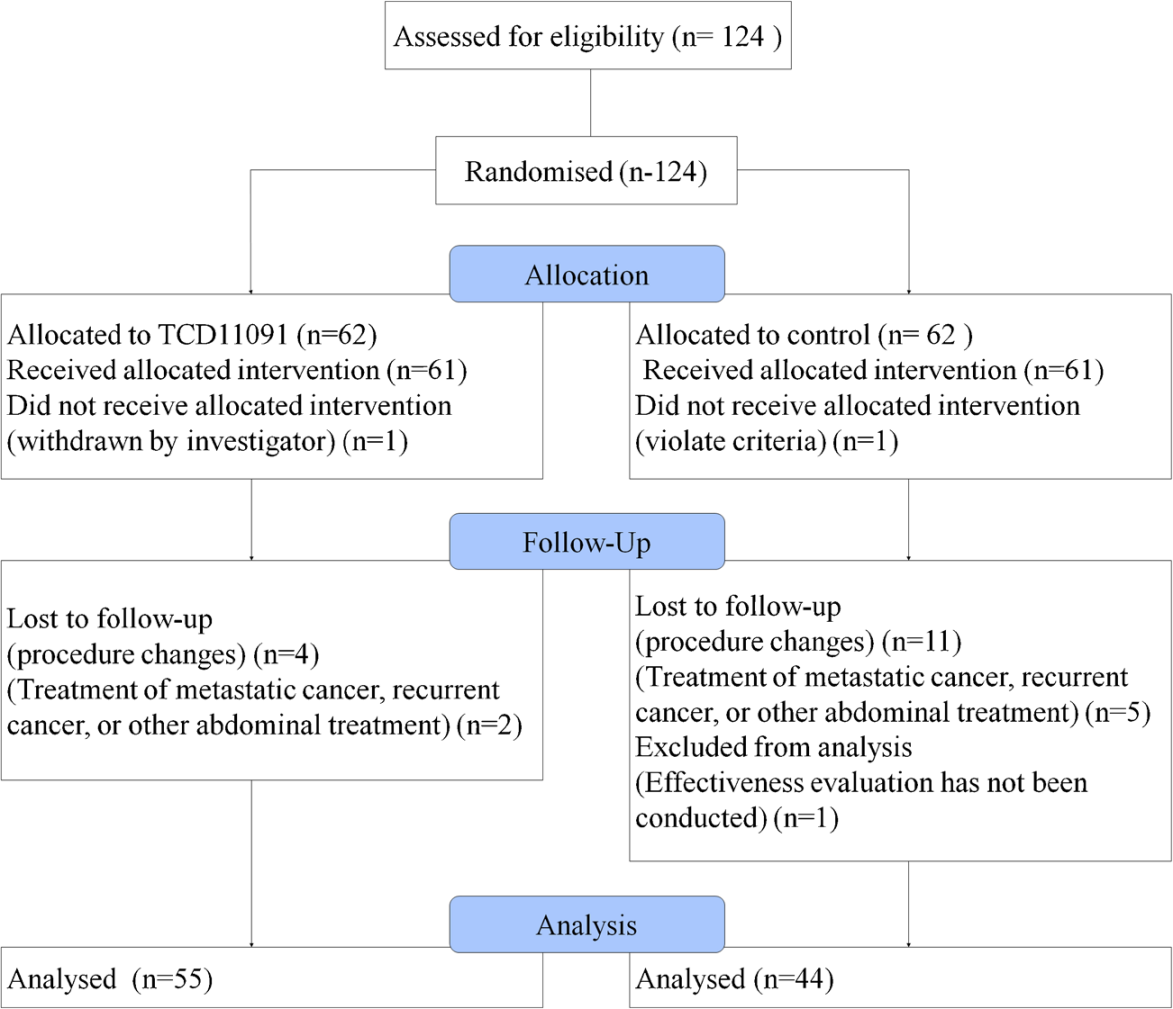
Patient flow chart

A total of 124 patients were enrolled in this study between December 2012 and June 2014. Patients were allocated to the TCD-11091 group or the control group in a 1:1 ratio: 62 patients were allocated to both the TCD-11091 group and the control group. No malfunction of TCD-11091 occurred during this study. TCD-11091 contained 4.9 mL adhesion barrier gel per one kit. TCD-11091 was used 2.9 ± 0.6 kit per patient. TCD-11091 was used to cover the organs under the median incision, and the coverage of the organ was confirmed by investigator. The numbers of patients who completed the assessment after the second-look surgery were 55 in the TCD-11091 group and 44 in the control group. The breakdown of withdrawals who did not undergo assessment after the second-look surgery is as follows: one patient (the control group) was found to have a history of intraperitoneal surgery before the initial surgery and met the exclusion criteria, one patient (the TCD-11091 group) was withdrawn by the investigator, 15 patients (four in the TCD-11091 group and 11 in the control group) were withdrawn because of a change in the surgical procedure during the initial surgery, seven patients (two in the TCD-11091 group and five in the control group) put priority on treatment of metastatic cancer or recurrent cancer or other treatments including laparotomy before the second-look surgery, and one patient (the control group) could not undergo an efficacy evaluation since investigator could not observe the organ under the median incision by laparoscope. Withdrawal from the study was determined based on the medical findings with consideration given to the postoperative and prognostic safety of and risks to the patients. The patient flow chart is shown in Fig. 1.

A background data including medical histories and tumor stage are shown in Table 4. The primary disease in almost all patients was rectal cancer (98.4 and 96.7%), and the other patients had rectal carcinoid tumors with a stoma. No significant differences in age, sex, body mass index, history of intestinal disease, history of surgery, and complicated diseases such as hypertension, hyperlipidemia, diabetes mellitus, and hyperuricemia were found between the TCD-11091 group and the control group. There were no significant differences in the length of midline incision of lower abdomen, intraoperative blood loss and operative time during primary surgery, and the frequency of adjuvant chemotherapy in both groups.

**Table 4:** Patient background

Items		TCD-11091		Control		*P* value
		*N*	% (*n*/*N*)	*N*	% (*n*/*N*)	
Total patients		61		61		
Sex (male)		51	83.6%	47	77.0%	0.362^a^
Age		60.5 ± 9.9		62.3 ± 10.2		0.341^c^
BMI		23.05 ± 3.25		22.70 ± 3.26		0.556^c^
TNM stage	0	0	0.0%	0	0.0%	0.946^b^
	I	14	23.0%	10	16.4%	
	IIA	13	21.3%	22	36.1%	
	IIB	0	0.0%	0	0.0%	
	IIIA	4	6.6%	2	3.3%	
	IIIB	18	29.5%	13	21.3%	
	IIIC	11	18.0%	12	19.7%	
	IV	0	0.0%	1	1.6%	
History of intestinal disease		7	11.5%	9	14.8%	0.592^a^
History of surgery		8	13.1%	9	14.8%	0.794^a^
Complications		49	80.3%	54	88.5%	0.212^a^
	Hypertension	26	42.6%	22	36.1%	
	Hyperlipidemia	6	9.8%	9	14.8%	
	Diabetes mellitus	11	18.0%	11	18.0%	
	Hyperuricemia	5	8.2%	6	9.8%	
	Others	44	72.1%	39	63.9%	
Length of midline incision (cm)		20.22 ± 5.51		19.71 ± 4.78		0.605^c^
Intraoperative blood loss of primary surgery (ml)		552.3 ± 503.6		736.7 ± 838.0		0.162^c^
Operative time of primary surgery (min)		438.8 ± 119.4		451.5 ± 109.1		0.560^c^
Neoadjuvant chemotherapy		8	13.1%	3	4.9%	0.114^a^

^a^ *χ* ^2^ test

^b^Wilcoxon rank sum test

^c^ *T* test

### Efficacy

**Fig. 2:**
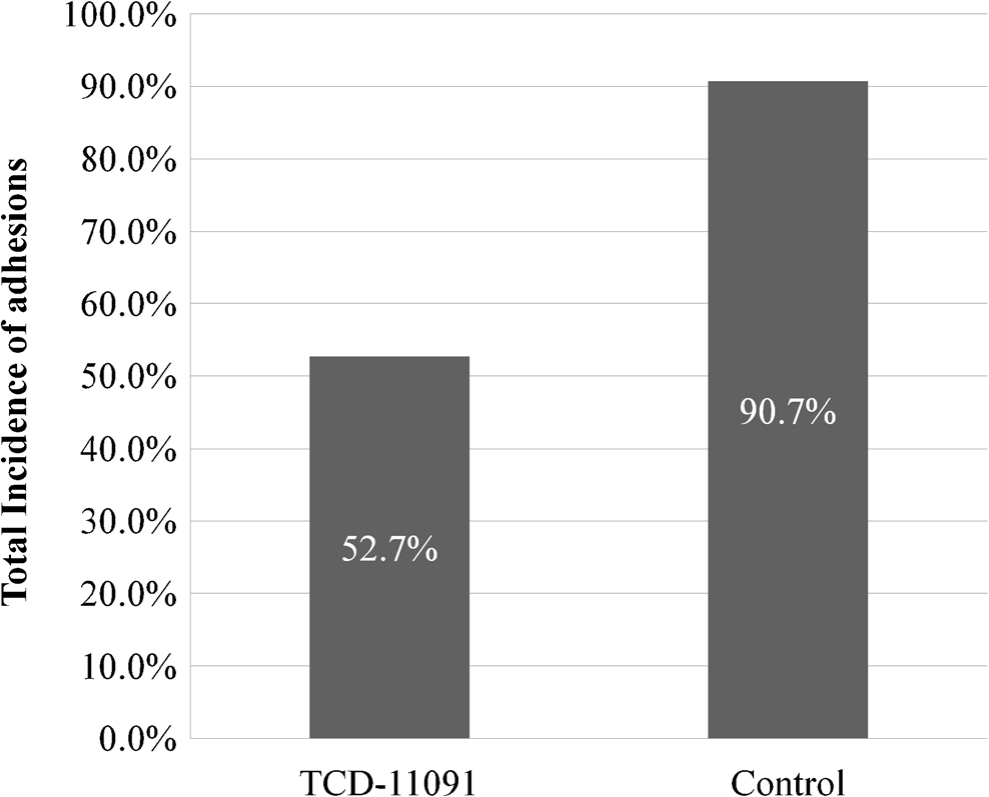
Incidence of adhesions

**Fig. 3:**
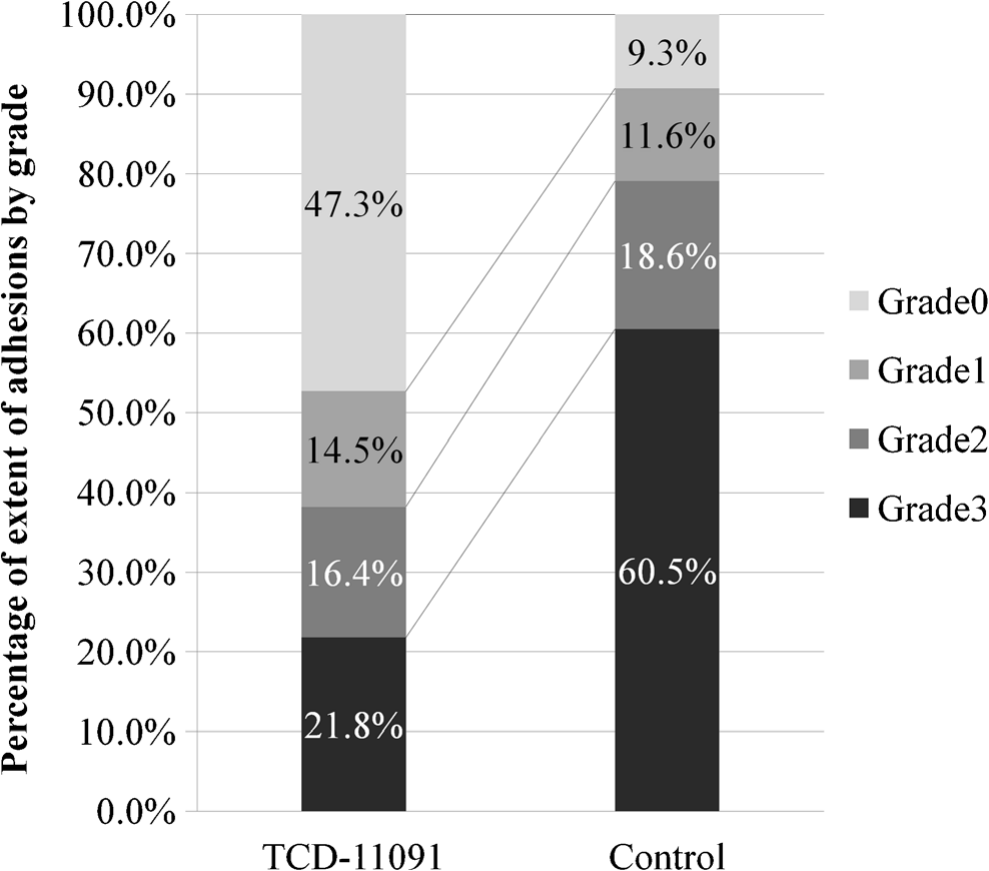
Extent of adhesions

**Fig. 4:**
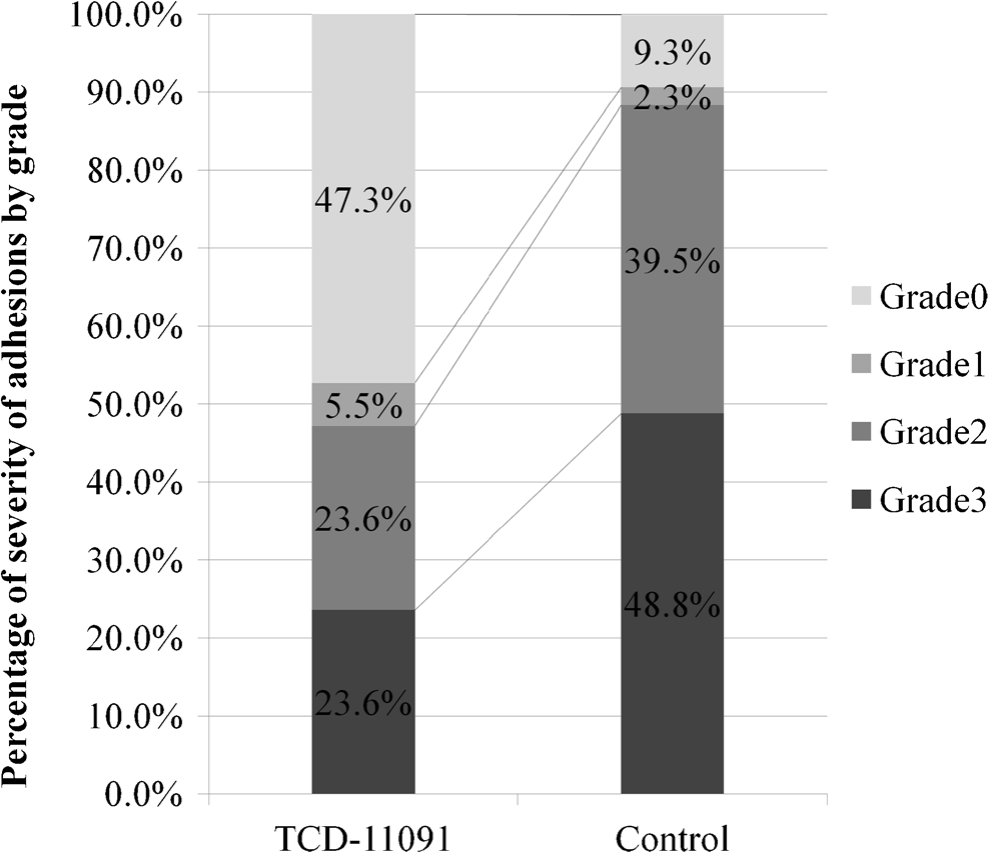
Severity of adhesions

The incidences of adhesions under the midline incision, the primary endpoint, were 52.7% in the TCD-11091 group (29/55) and 90.7% in the control group (39/43); the incidence of adhesions in the TCD-11091 group was significantly reduced compared with that in the control group (*p* < 0.001) (Fig. 2). For the secondary endpoints, adhesions of wide extent (grade 2 or higher) occurred 38.2% (21/55) and 79.1% (34/43); the incidence of wide extent adhesions was significantly reduced (*p* < 0.001) (Fig. 3). In terms of adhesion severity, grade 2 or higher adhesions were noted 47.3% (26/55) and 88.4% (38/43); the severity of adhesions was significantly reduced by TCD-11091 (*p* < 0.001) (Fig. 4).

### Safety

The incidences of adverse events were 85.2% in the TCD-11091 group (52/61) and 75.4% in the control group (46/61); no significant difference was noted (*p* = 0.225). Adverse events that occurred at a relatively high frequency were “pyrexia,” “peripheral neuropathy,” “intestinal obstruction,” “nausea,” and “palmar-plantar erythrodysaesthesia syndrome.” The incidences of MAEs were 21.3% (13/61) and 14.8% (9/61); no significant difference was noted (*p* = 0.360). The frequently reported MAE was functional intestinal obstruction: 11.5% (7/61) and 9.8% (6/61); no significant difference was noted (*p* = 0.779). Serious adverse events were reported in 26.2% of the patients (16/61) and 23.0% (14/61); no significant difference was noted. There were no adverse events that resulted in death or were life-threatening. No cases of carcinogenesis or infections related to TCD-11091 were reported. The success rate for the use of the TCD-11091 was 93.0% (53/57). A total of four cases (five kits) were considered unsuccessful. However, these cases were not considered to meet the criteria for malfunction because all of them were caused by misuse of the device (Table 5).

**Table 5:** Breakdown of major adverse events

	TCD-11091		Control		*P* value^a^
	%	*n*/*N*	%	*n*/*N*	
Total	21.3	13/61	14.8	9/61	0.481
Functional intestinal obstruction	11.5	7/61	9.8	6/61	1.000
Mechanical intestinal obstruction	6.6	4/61	3.3	2/61	0.680
Abdominal (abdominal cavity) abscess	1.6	1/61	0.0	0/61	1.000
Peritonitis	1.6	1/61	0.0	0/61	1.000
Postoperative wound infection	1.6	1/61	1.6	1/61	1.000

^a^Fisher’s exact test

## Discussion

This study was conducted in patients with primary rectal cancer who were scheduled to undergo laparotomy with a loop ileostomy in order to confirm that the incidence of adhesions is reduced significantly in TCD-11091-treated patients compared with non-treated patients and also to confirm the safety of the study device. To determine adhesion status, adhesions under the midline abdominal incision were observed directly through a laparoscope at the time of ileostomy closure (second-look surgery) and assessed by the independent efficacy assessment committee, thereby securing the objectivity of assessment. The presence, extent, and severity of adhesions were assessed by the efficacy assessment committee based on the video images taken using the standardized procedure at the time of the second-look surgery. These assessment criteria for adhesions were established based on the adhesion scores of the Re-ASRM classification,^7^ which are used in laparoscopic diagnosis of endometriosis. The assessment criteria used in previous clinical studies of adhesion barrier materials were also referred to.^3^, ^4^, ^8^, ^9^

In the control group, the incidence of adhesions was 90% or higher, and many adhesions were classified as higher grades. In contrast, in the TCD-11091 group, the incidence of adhesions was approximately 50%, and the extent and severity of adhesions were reduced significantly. Therefore, the efficacy of TCD-11091 as an adhesion barrier material was demonstrated. Regarding safety, no significant differences were noted between the TCD-11091 and non-treated groups in terms of the incidence of adverse events, serious adverse events, and major adverse events; the safety of TCD-11091 was also considered to be unequivocally acceptable. Consequently, the efficacy and safety of TCD-11091 were confirmed, and the usefulness of TCD-11091 as a new adhesion barrier material was demonstrated.

In this study, the incidence of adhesions in TCD-11091-treated patients was reduced to approximately half (50%) that (90%) of non-treated patients. As indicated by the incidence of adhesions, the treatment outcomes in this study were virtually the same as the previous five studies (incidences of 86.2 to 100%)^3^–^6^, ^8^ as well as that reported by Becker et al.^8^ The surfaces of the organs in the abdominal cavity are covered by the peritoneum consisting of the mesothelium, basement membrane, and connective tissues. The peritoneum continuously maintains a wet state that reduces friction and supports the activities of the organs. It is considered that if the peritoneum is injured and more fibrin is released than can be absorbed, fibrin networks form among the organs, which are then more likely to form fibrous adhesions. The surgical procedures performed in this study may cause more bleeding than other gastrointestinal forms of surgery such as colon cancer resection and partial gastrectomy, and include lymph node dissection in the pelvis. Therefore, the procedures are highly tissue-invasive, prolonged, and affected by the continuous exudation of fibrin and lymph as well as drying damage caused by exposure of the peritoneum. Collectively, this generates many risk factors that can lead to the formation of adhesions. In the control group, the incidence of adhesions was 90% or higher and many cases were classified as higher grades in terms of both the extent and severity of adhesions. Under these conditions, the use of TCD-11091 suppressed the incidence of adhesions to approximately 50% and also reduced their extent and severity. These results show that TCD-11091 can be expected to act effectively as an adhesion barrier material in patients who undergo highly invasive surgery.

Intestinal obstruction that is due to adhesions in the abdominal cavity is a significant adverse event in terms of its seriousness. The incidence of adhesive intestinal obstruction classified as mechanical intestinal obstruction was 6.6% (4/61) in the TCD-11091 group and 3.3% (2/61) in the control group (*p* = 0.680). The incidence of functional intestinal obstruction caused by stagnation of bowel contents resulting from reduced intestinal movement in association with intestinal neuropathy was 11.5% (7/61) and 9.8% (6/61) (*p* = 0.779). There were no significant differences between the groups either in mechanical intestinal obstruction or in functional intestinal obstruction. In this study, the location of adhesive intestinal obstruction could not be clearly identified. The use of the study device was limited to organs under the midline incision. Therefore, it could not be clearly determined whether adhesive intestinal obstruction occurred inside or outside the area in which the study device was used.

The incidences of intestinal obstruction in this study were 18.0% (11/61) in the TCD-11091 group and 13.1% (8/61) in the control group; no significant difference was noted (*p* = 0.618). The occurrence of adhesive intestinal obstruction and the results of efficacy evaluation were not completely consistent in the TCD-11091 group. Nevertheless, it is not thought that TCD-11091 was responsible for inducing intestinal obstruction.

The bio-absorbable and anti-adhesive material used in this study is a spray-type adhesion barrier material. The advantages of the material are easy handling, suitability for a wide range of procedures and treatment sites, and easy application within a confined space, such as in the pelvic cavity. This ease of use is achieved by means of the nozzle and the use of a trocar now commonly employed in laparoscopic procedures. The use of TCD-11091 in the treatment of various conditions is expected to reduce the occurrence of adhesions in the abdominal cavity of patients undergoing surgery in the future, thereby leading to improved surgical outcomes.

## Conclusion

This prospective randomized single-blind study demonstrated that use of TCD-11091 was safe and associated with significantly lower incidence of adhesion and severe adhesion compared with non-treatment procedure.

## Limitation

This study was a prospective randomized study with control patients as controls. However, with only 124 patients participating, the total sample size was small. TCD-11091 was applied to the organs under the midline incision of the abdomen in patients with primary rectal cancer who underwent temporary ileostomy. Therefore, there were withdrawals from the study due to changes in the surgical procedure, cancer recurrence resulting from medical conditions of the cancer itself, and adverse events related to the rectal cancer surgery itself. In the future, it would be desirable to assess the treatment outcomes of other procedures in the abdominal cavity and pelvic cavity as well as those requiring approaches, such as laparoscopic surgery using a trocar or a small incision.
